# Continuing a Cancer Treatment Despite Tumor Growth May Be Valuable: Sunitinib in Renal Cell Carcinoma as Example

**DOI:** 10.1371/journal.pone.0096316

**Published:** 2014-05-05

**Authors:** Mauricio Burotto, Julia Wilkerson, Wilfred Stein, Robert Motzer, Susan Bates, Tito Fojo

**Affiliations:** 1 Medical Oncology Branch, Center for Cancer Research, NCI, NIH, Bethesda, Maryland, United States of America; 2 Hebrew University, Jerusalem, Israel; 3 Memorial Sloan Kettering Cancer Institute, New York, New York, United States of America; Davidoff Center, Israel

## Abstract

**Background:**

The US FDA and the EMA have approved seven agents for the treatment of renal cell carcinoma, primarily based on differences in progression-free survival (PFS). Because PFS is an arbitrary endpoint we hypothesized that an analysis would demonstrate the growth rate of tumors remained constant at the time of RECIST-defined disease progression.

**Methods:**

We previously estimated the growth (**g**) and regression (**d**) rates and the stability of **g** using data from the Phase III trial comparing sunitinib and interferon.

**Results:**

Sufficient data were available and rate constants statistically valid in 321 of 374 patients randomized to sunitinib. Median **d** was 0•0052 days^−1^; in 53 patients no tumor growth was recorded. Median **g** was 0•00082 days^-1^ and was stable for a median of 275 days on therapy, remaining stable beyond 300, 600 and 900 days in 122, 65 and 27 patients, respectively. A possible increase in **g** while receiving sunitinib could be discerned in only 18 of 321 patients. Given a median **g** of 0•00082 days^−1^ the estimated median time to a second progression were sunitinib continued past RECIST-defined progression was 7.3 months. At 100, 200, and 300 days after starting therapy, an estimated 47%, 27%, and 13% of tumor remains sunitinib sensitive and could explain a RECIST-defined response to a new TKI.

**Conclusion:**

Prolonged stability of **g** with sunitinib suggests continued sunitinib beyond RECIST-defined progression may provide a beneficial outcome. Randomized trials in patients whose disease has “progressed” on sunitinib are needed to test this hypothesis.

## Introduction

In the last seven years the U.S. Food and Drug Administration (FDA) and the European Medicines Agency (EMA) have approved seven agents for the treatment of advanced renal cell cancer (RCC). [1]–[6] Five of these agents target the VEGF pathway while two target the mammalian target of rapamycin (mTOR). The availability of so many agents means that in the treatment of metastatic RCC there are many different options, whether in first or second line after progression that must be properly evaluated.

The Response Evaluation Criteria in Solid Tumors (RECIST) assessment criteria, often used as a guide to quantify progression in clinical trials, have provided investigators a language to communicate clinical trial outcomes. [7] While an increase in the sum of the longest diameters by 20% meets RECIST criteria for progression, there is no clinical evidence that this quantity is a clinically valid endpoint that should result in a change of therapy. Similarly there is a lack of solid evidence supporting the use of drugs with an apparently similar target, such as VEGFR, in succession – an area in need of investigation since many of the therapies approved for RCC have similar targets.

We have previously demonstrated that the rate of growth and regression of tumors can be determined using tumor measurements obtained during the course of treatment. [8]–[11] In the present study, using data from the Phase III trial that compared sunitinib and interferon, [2] we demonstrate the stability of the rate of growth during treatment with sunitinib, and model, using the median rate of growth, the outcomes expected after RECIST-defined progression is documented. Using the estimated values for the rate of growth of RCC while on sunitinib, we demonstrate that continued sunitinib could be a valid alternative following RECIST-defined progression on sunitinib.

## Methods

We conducted a detailed analysis of data from the sunitinib registration trial examining the growth (g) and regression (d) rates and the stability of the growth rate as measures of effectiveness and to understand development of resistance.

The institutional review board of all participating centers involved in the trial #NCT00083889 approved the original study, and all patients signed informed consent. Prior to the present analysis patient's information was anonymized and de-identified. For the analyses presented in the study, anonymized tumor measurement data, enrollment and off-study dates, and date of death data were provided in spreadsheet format by Pfizer, Inc without any restrictions. The National Institutes of Health/‘National Cancer Institute provided authorization for this analysis.

### Clinical trial and study design

The study, an international, multicenter, randomized, phase III trial, compared sunitinib (Sutent®, Pfizer), with interferon alfa (IFN-α). Results, as well as details of the design of this trial have been previously published. [2], [12] Tumor measurements from CT scans were recorded as the sum of longest diameter (LD) of target lesions. Responses and progressions were assessed according to Response Evaluation Criteria in Solid Tumors (RECIST 1.0). Growth rate constants derived from these data have been reported, confirming a greater reduction in the growth rate constant **g**, for sunitinib compared to that obtained for IFN-α. [11]

### Mathematical, data, and statistical analyses

#### Mathematical analysis

Our regression-growth equation is based on the assumption that change in tumor quantity during therapy, indicated by change in the sum of LDs, results from 2 independent component processes (both following first order kinetics): an exponential decrease/regression, **d**, and an exponential growth/regrowth of the tumor, **g**: [8]–[11]



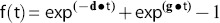
(1)where exp is the base of the natural logarithms, e = 2.7182…, and f(t) is the tumor quantity (f, sum of LDs) measurement at time t (days), normalized to (divided by) the tumor quantity at day 0, when treatment commenced. During therapy, rate constant **d** (**d**ecay, in days^−1^) characterizes the exponential decrease/regression of the tumor, and rate constant **g** (**g**rowth, in days^−1^) the exponential growth/re-growth of the tumor.

When the data show a continuous decrease from the start, only the regression parameter **d** differs significantly from zero with p<0.1, and Eq(1) is replaced by:



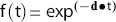
(2)Likewise, when tumor measurements show a continuous increase, only the growth parameter **g** differs significantly from zero with p<0.1, so Eq(1) is replaced by:



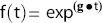
(3)


Finally, these rate constants may be expressed in terms of half-lives and doubling times. Thus, the regression rate, **d**, equals ln2 ( = 0.693.) divided by the time it takes for the regressing part to shrink by half, while the growth rate, **g**, equals ln2 divided by the time for the growing component to double (doubling time).

#### Statistical Analysis

Analysis and output was generated using Base SAS and SAS/STAT software, Version 9.1.3 of the SAS System for Windows (SAS Institute Inc., Cary, NC). Non-linear regression using the least squares method was performed for Eqs(1–3) in all patient data sets with ≥2 evaluations [Eq(1) in all patient data sets with ≥3 data points, Eqs(2–3) in all patient data sets with ≥2 data points. Parameter estimates, standard errors, t and p-values were output. Model selection was applied in the following order for cases with more than two evaluations (level of significance set at 0.1): (i) Eq(1) selected where both parameter estimates (**g, d**) were significant. (ii) Eqs(2–3) where only the single parameter (**d** or **g**) was significant. In the latter single parameter cases, the complementary parameter was arbitrarily set at 0. For cases with only two data evaluations, parameter estimates from Eqs(2–3) were included only where the ratio from the start value was ≤0.80, or ≥1.20 respectively. We have used the latter when analyzing radiographic data in solid tumors to coincide with RECIST allowances for variability in measurements and maintained that paradigm here.

## Results

**Figure 1:**
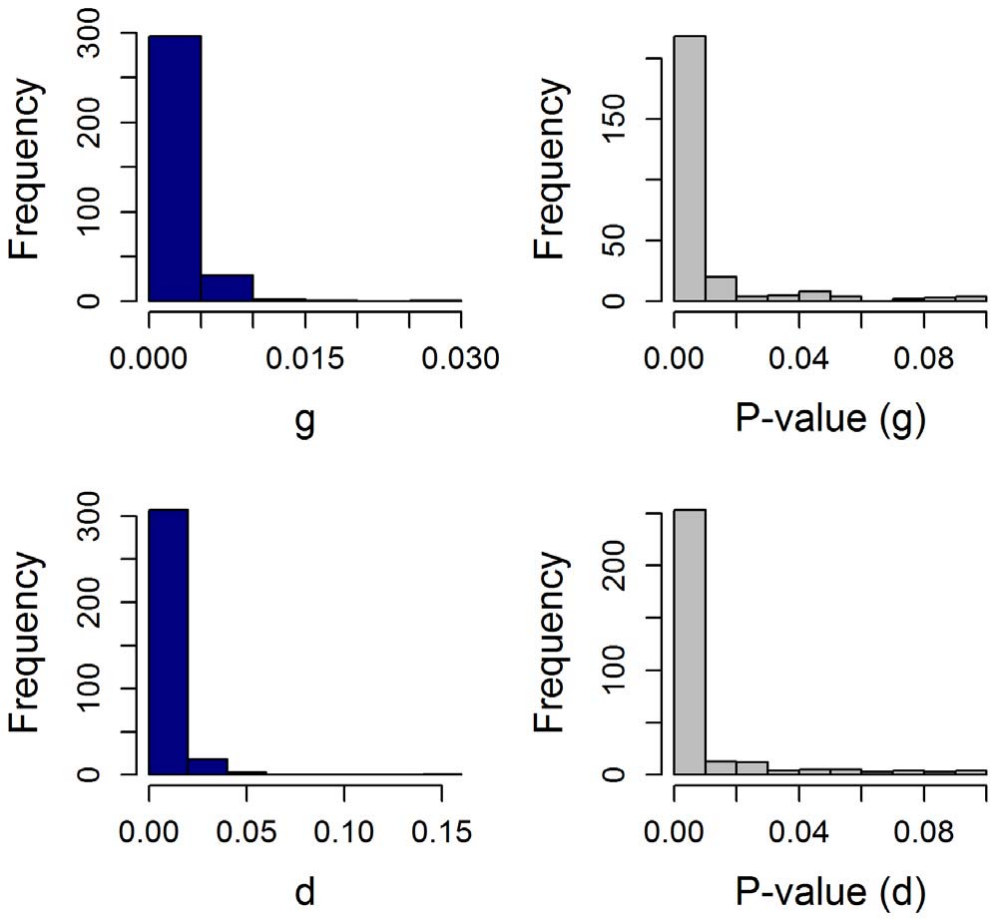
Histograms depicting the distributions of the g and d values and the corresponding regressor p-values. While a p value of <0.1 was accepted as valid in this analysis, in fact as can be seen, the overwhelming number of p-values were much lower than that, an indication of the validity of the fits [median g = 0.00084; median p-value for g = 0.000047; median d = 0.0050; median p-value for d = 0.00024]. Note the expected higher value for median d compared to median g. See also **File S1**.

For this analysis we utilized the sunitinib data from the clinical trial that randomized patients to receive either sunitinib or interferon alfa (IFN-α) and that was used to support the registration of sunitinib for the treatment of RCC. [2] Three hundred and seventy-four patients were randomized to receive sunitinib. Sufficient data were available to perform the analysis for 350/374 patients randomized to sunitinib. [11] The reasons for the exclusion of twenty four subjects due to insufficient data were the following: one with no data, six with only one data point and seventeen with only two data points that differed by less than 20% and thus would not have met RECIST criteria for either progression or regression. Setting the significance for the estimated parameters at *P*<0.1, valid results were obtained in 321 (91.7%) of the 350 patients with data sufficient for analysis. In the other 29, the data was not fit by any of the models (p
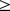
0.10). [11] Histograms depicting the distributions of the g and d values and their corresponding p values as measure of the probability of the regressors are shown in **Figure 1** **(See also File S1)**.

**Figure 2:**
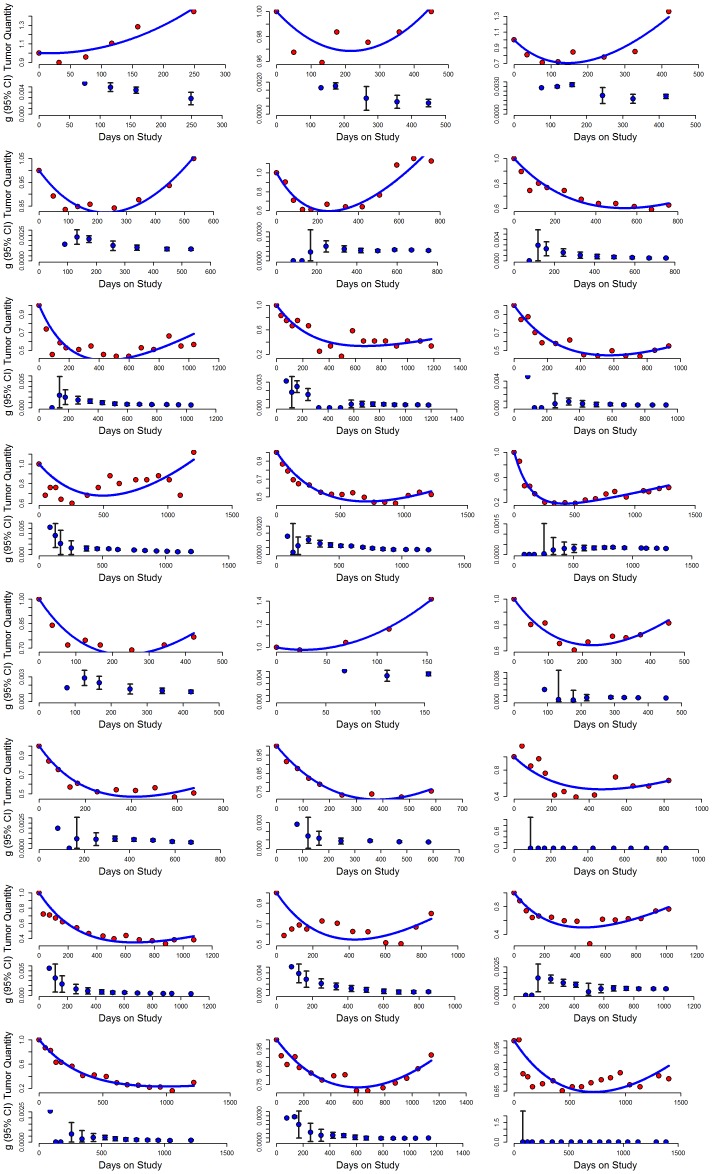
Sigmaplot curve fits of tumor quantity over time in 24 patients randomized to sunitinib in the registration trial. The majority of patients randomized to sunitinib had no evidence of acceleration in the rate of growth for hundreds of days. The majority had stable rates of growth as shown above; some had only evidence of tumor regression, but these are not shown. In each example, the upper graph plots the observed tumor quantity measurements obtained by the clinical investigators during the patient's participation in the clinical trial as well as the predicted values from the best-fit model. The lower panel of each pair depicts the growth rate constant, g, calculated with the data gathered up to that point in time, showing serial calculations of this value. The first calculation is done when three data points had been obtained, and each point thereafter depicts the estimate and 95% CI of the growth rate constant as each new data point is obtained.

**Figure 3:**
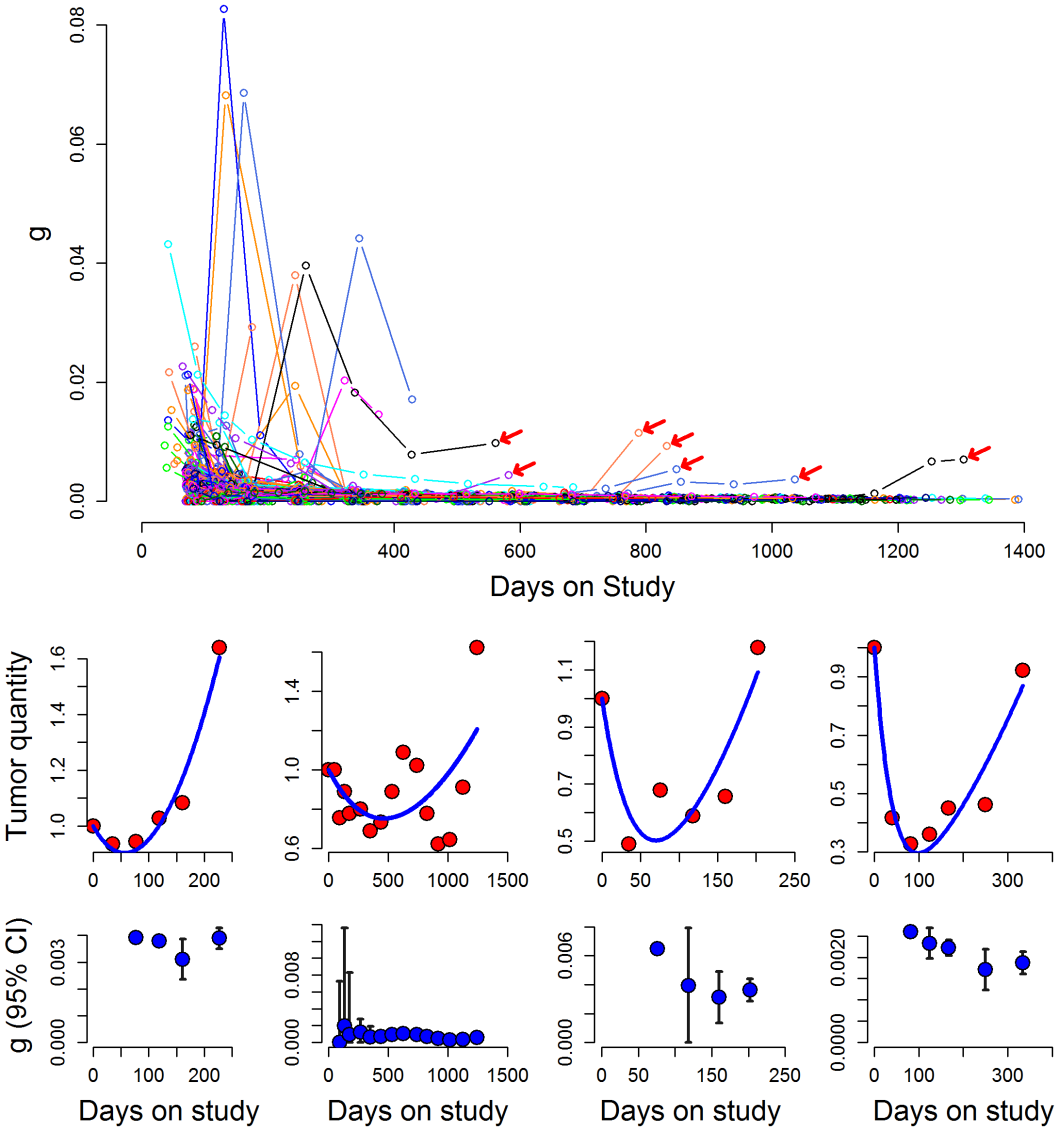
Plot showing the growth rate over time for the 234 patients who had any evidence of tumor growth. Tumor growth rates remained stable in the overwhelming majority. Red arrows point to examples of some of the 18 in whom there was an increase. Four of these are shown in the bottom panel.

The median regression rate constant was 0.0052 days^−1^, and in 53 patients no evidence of growth was recorded *while on study*, only regression. The majority of these patients likely discontinued drug treatment because of toxicity or because it was felt that maximum benefit had been achieved but may have experienced progression had they continued on treatment. The median growth rate for the 321 patients with a valid fit of their data was 0.00082 days^−1^. [11] **Figure 2** depicts 24 examples from several hundred similar cases, chosen to represent the sample population. In each example, the upper graph plots the observed tumor quantity measurements obtained by the clinical investigators during the patient's participation in the clinical trial plotted according to the best-fit model. We would emphasize that the observed values are the actual values (sum of LDs) obtained for the patient and are displayed as the amount of tumor relative to a quantity of 1 (one) at enrollment. The lower panel of each pair depicts serial estimates of the growth rate constant, g, estimated with the data available at each point in time (i.e., the first calculation uses the first three data points, and each point thereafter depicts the new estimated g and 95% CI with the addition of each new data point). The examples include data from patients who received treatment for a long time, allowing one to appreciate the stability of the growth rate constant. Note how in a given patient as additional points are added the symbols and their confidence interval remain largely unchanged, and overlap, this despite the sensitivity of the Y-axes. Indeed, after a median of 117 days (a median of 4 data points) we could obtain an estimate of g for each patient that was not statistically different from the value with the entire data set. This outcome was not unexpected given the interval between assessments. Importantly, once this statistically valid value was achieved, the rate remained stable a median of 275 days, remaining stable beyond 300, 600 and 900 days in the 122, 65 and 27 patients with data available until these time points. A suggestion of a possible increase of the growth rate while sunitinib was administered could be visually discerned in only 18 of the 321 patients. This result is depicted in **Figure 3** which plots the serial estimated growth rate constants over time for the 234 cases in which both an estimate of g was obtained (p<0·10) and there were three or more data points – it does not include the fifty-three patients in whom a g could not be estimated, nor 34 patients with three or fewer data points. As can be seen, an increase of the growth rate (g) was rarely observed and did not occur with greater frequency over time – with arrows indicating examples of *some* of those in whom there was some evidence to suggest an increase or acceleration. The bottom panel of **Figure 3** shows four examples where the rate of growth (g) was possibly increasing. These results underscore the fact that while the tumors in most patients were growing, albeit at varying rates while receiving sunitinib, the growth rates in the large majority were constant and not increasing. Lacking any evidence of such an increase, one can then estimate how long it would have taken at the “on-sunitinib rate” to reach a value 1.2 fold above that measured at any point in time – the *minimum* amount of increase needed to qualify for disease progression according to the RECIST criteria. With a median growth rate 0.00082 days^−1^ the estimated time to a second progression, from the point of the first progression, would have been a minimum of 7.3 months. [ln 1·2/0.00082 days^−1^ = 0.1823/0.00082 days^−1^ = 222 days = 7.3 months ]. This then is the estimate of the time that would elapse before RECIST progression would be scored provided continued sunitinib is tolerable. This value can be compared with the results that have been achieved with agents used in second line as shown in **Table 1**.

**Table 1:** Survival endpoints in second line in patients treated in first line with sunitinib.

Drug	Total number [Prior sunitinib subgroup]	PFS	OS	Author
Everolimus	277 [124]	3.9 months	14.8 months	Calvo 2012 [26]
Sorafenib	362 [195]	3.4 months	19.2 months	Rini 2011 [13]
Axitinib	361 [194]	4.8 months	20.1 months	Rini 2011 [13]

PFS, progression free survival; OS, overall survival.

**Figure 4:**
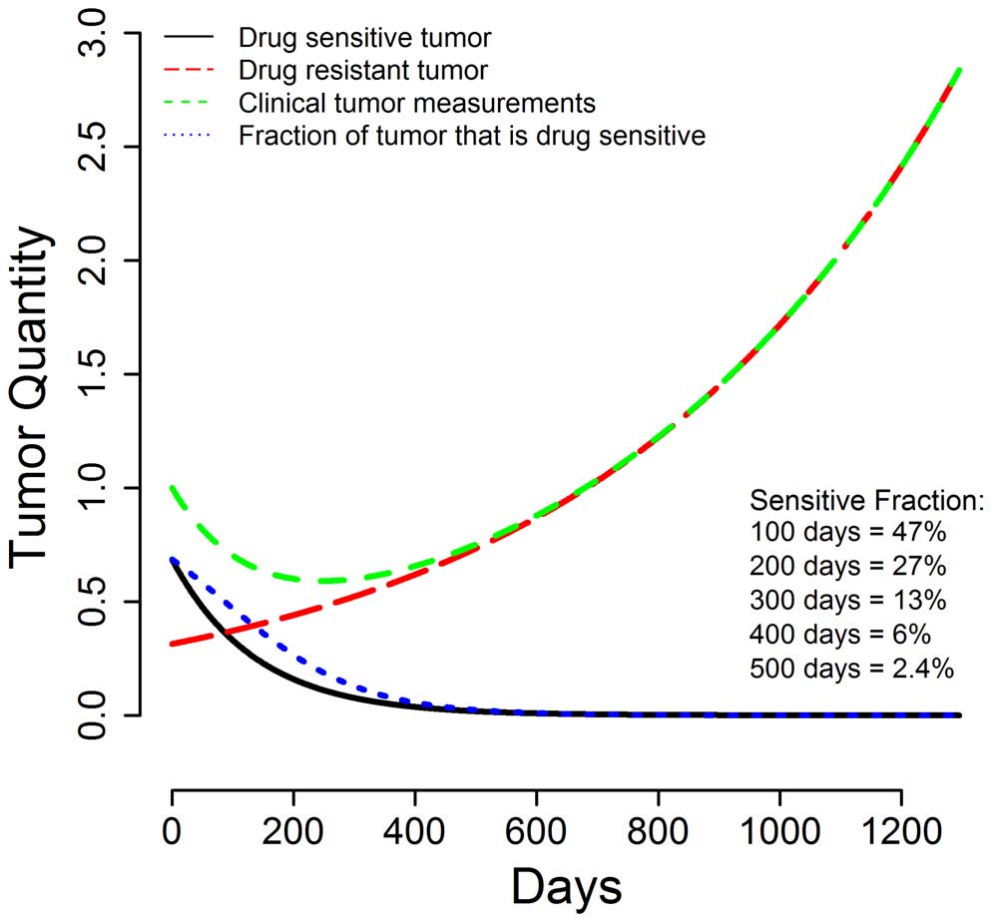
Graph depicting estimates of the fraction of the remaining tumor that is sensitive to a therapy at various times during its treatment. If a therapy is discontinued prematurely for a reason other than progressive disease, evidence of tumor shrinkage with a second line option may not be indicative of non-cross resistance since not all sensitive tumor will have been killed. The graph depicts curves drawn using the median g (0.00084) and d (0.0050) values of all patients enrolled in the sunitnib arm of the study and shows: (a) the actual tumor measurements as sum of the LDs (green line); (b) the gradual decrease in the fraction of tumor that is sensitive to therapy (black line); (c) the gradual increase in the fraction of tumor relatively resistant to therapy (red line); and (d) the fraction of the remaining tumor that is still sensitive to the therapy that is being administered (blue line). Were therapy stopped before all tumor sensitive to that therapy has been eliminated, tumor shrinkage could occur even though the new therapy is not “different”.

Finally, using the median estimated growth (g) and regression (d) rate constants one can draw theoretical curves depicting tumor quantity over time as shown in **Figure 4**. The green line is the clinically observed tumor measurement (sum of LDs), composed of the sensitive tumor quantity that is regressing (or decaying, black line) and the resistant tumor quantity that is growing (red line). [8]–[11] This allows one to estimate the fraction of tumor at any given point in time still sensitive to the therapy that is being administered (blue line):
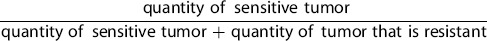
As can be seen 100, 200 and 300 days after starting therapy an estimated 47%, 27% and 13% of tumor is still sensitive to sunitinib. Thus in patients who discontinue sunitinib before day 300 for a reason other than progressive disease and receive a new therapy, tumor shrinkage cannot be considered unequivocal evidence of non-cross resistance. The remaining sensitive fraction could result in sufficient shrinkage to qualify as an objective or minor response and be depicted as measurable shrinkage on a waterfall plot. But as **Figure 4** shows, this outcome could be simply a result of residual sensitive tumor.

## Discussion

We report the results of an analysis of the growth rate of renal cell carcinoma (RCC) while sunitinib was administered. We have previously reported that compared to interferon alfa, sunitinib was able to more effectively reduce the growth rate of renal cell carcinoma and that this reduction was correlated with better progression-free and overall survival and was likely responsible for the greater efficacy of sunitinib. [11] But because sunitinib does not eradicate all tumors, disease recurrence is eventually expected to occur. We demonstrate here prolonged stability of the growth rate of RCC while sunitinib was administered and make predictions as to how effective continued sunitinib therapy might be. We believe the results indicate that, barring toxicity, continued sunitinib beyond RECIST criteria for progression could provide a beneficial outcome. These analyses suggest that randomized trials assessing the value of salvage therapies in patients whose RCC has met disease progression criteria on sunitinib or a similarly effective agent should consider including an arm that continues sunitinib or the similarly effective agent to test the possibility that continued treatment might be as or more beneficial than changing to a salvage therapy. Today, many view axitinib as the standard of care in second line RCC, based on a randomized trial that compared sorafenib with axitinib as second line therapy. [13] In this trial PFS was 6.7 and 4.7 months in the axitinib and sorafenib arm, respectively (P<0.0001). Both the FDA and the EMA approved axitinib as a second line alternative based on these results. Updated results of this trial did not find a statistically significant difference in OS.[14]

Progression-free survival, an increasingly common endpoint in registration trials, is thought to reflect a drug's efficacy. According to RECIST criteria, progression is reached when the quantity of tumor exceeds a value 20% above the nadir. In patients in whom regression occurs, PFS occurs after the initial fall in tumor quantity reaches its nadir and then increases 20%. The use of PFS as a surrogate for overall survival has been a contentious endpoint that has been discussed in depth. [15], [16] In contrast the underlying assumption that the 20% progression criterion indicates drug resistance and treatment failure has received less scrutiny. [16]–[19] If an agent were not eradicating the tumor but instead were continuously slowing tumor growth while administered, the 20% threshold could represent an artificial boundary – especially if no other therapy can bring about a cure.

By estimating the growth (**g**) and regression (**d**) rate constants we are able to dissect into its regression and regrowth components the commonly recorded observation of an initial tumor regression that after reaching a nadir is followed by regrowth of the tumor. This allows us to predict future outcomes. In the majority of cases, the tumor that is growing is not *absolutely resistant* to the therapy, but only *relatively resistant* since the administered therapy is having some effect that is manifested as slowing of growth. Furthermore, as **Figure 4** shows, the sensitive fraction is eventually virtually eliminated and only this relatively resistant fraction remains. Importantly, the data here, and indeed in other malignancies that we have examined (unpublished observations) demonstrate that the rate of growth of this relatively resistant tumor in most patients remains constant without evidence of acceleration. We would note here that we are focusing on a rate of increase rather than on the absolute increase in tumor quantity. That is to say, while a tumor that is being measured at three month intervals may increase at a constant rate from 1 to 2 to 4 to 8 to 16 “quantity of tumor” and so on – a doubling every three months - the absolute increase in tumor quantity is obviously getting larger over time. But an effective drug may prolong that doubling time to six months. It is the constancy of the growth rate that allows one to accurately predict what the next quantities (measurements) will be (32 and 64 in the example above). A further extrapolation allowed by this constancy is that we can predict when 120% of a new baseline would occur – that is to say when a second RECIST progression would be scored.

Should we be surprised from the standpoint of cell biology that the rate of increase remains constant? We would argue no, since this rate depends on many variables and not just one, and as long as the tumor is surviving therapy, there is no particular survival advantage to a more rapid growth rate. Also with many factors likely contributing to the growth rate, it is unlikely a change in just one would have much impact on the rate of growth.

While it is common to conduct clinical trials in second and subsequent lines of treatment that compare a given therapy against another or a placebo, it is rare to include an arm that continues the therapy on which progression has just been scored. The implication of the term progression is that treatment failure has now occurred and progression will occur again rapidly if therapy is not changed. Furthermore, in a practice setting, patients want to move on to the next therapy that they hope might cure them of their cancer. But given that the overwhelming majority of our therapies for metastatic solid tumors are not curative but only prolong life, a paradigm that looks to administer the most growth delaying therapy should be investigated. Assuming a therapy is tolerable and that it has slowed growth substantially, continuation of such a therapy should be an option. In this context, a projected PFS of 7.3 months with continued sunitinib would be a valid treatment option, competitive with current second line options that are summarized in **Table 1**.

We would also add that while this analysis has focused on sunitinib, we believe a similar analysis of other tyrosine kinase inhibitors targeting the VEGFR would lead to a similar conclusion. And given the limitations in developing countries where access to all drugs might not be possible or affordable, a paradigm shift that provides a rationale to continue an effective agent should be a welcome strategy. [20]

Finally, we would note that the paradigm of continuing treatment after “RECIST progression” has been explored in other tumors. For example, bevacizumab and trastuzumab have demonstrated efficacy “after progression” in metastatic colorectal and breast cancer respectively. [21], [22] While a retrospective analysis of patients whose tumors had progressed while treated with monotherapy erlotinib, a tyrosine kinase inhibitor used in non-small cell lung cancer, described the benefit of continued erlotinib with chemotherapy. [23] Additionally, ongoing clinical trials of crizotinib, a ALK kinase inhibitor, will formally test the finding of a preliminary report that argued for the feasibility and value of continued crizotinib administration. [24] Lastly, a phase III study in patients with metastatic gastrointestinal stromal tumors (GIST) whose disease had progressed on imatinib or sunitinib showed benefit when tumors were re-challenged with imatinib. [25] Our analysis is supported by these clinical studies and in turn provides a potential means for rationally selecting patients most likely to benefit from continuation of a given therapy.

In summary we present evidence that the growth rate of RCC treated with sunitinib remains stable over very prolonged periods of time and we predict that the drug would demonstrate efficacy if continued beyond RECIST-defined progression. We argue that continuation of an effective therapy such as sunitinib, which many consider the best first line option in RCC, may be an effective and possibly the most effective “second line” alternative. Lacking curative therapies for most solid tumors that have metastasized, our goal is to prolong life as long as possible. That goal may well be best achieved not by changing treatment but by continuing a therapy that is reducing the growth rate despite evidence of an increasing tumor burden.

## Supporting Information

File S1 **For the 24 cases displayed, the following diagnostic plots are provided from proc NLIN: a histogram of the raw residuals, a histogram of the projected residuals, a plot of observed versus predicted values, raw and projected residuals versus predicted values, standardized raw and projected residuals versus predicted values, raw residual expectation versus predicted values, standardized raw and projected residuals versus tangential leverage, standardized raw and projected residuals versus Jacobian leverage, a box plot of the raw and projected residuals, a leverage plot of tangential and Jacobian leverages versus observation number, a plot of local influence versus observation number, and a plot of raw and projected residuals versus time.** From these plots one can examine whether the model is a good fit in ways including: residuals appear randomly distributed (non-patterned) across the zero line, the plot of observed versus predicted values is closely and evenly distributed around the line plotted which has a slope of 1, and lack of observations showing super leverage, or leverage values in excess of 1. Additionally, both raw and projected residuals are displayed as the use of ordinary (raw) residuals (e = observed-predicted) for diagnostics of a nonlinear model that is intrinsically nonlinear can be misleading due to residuals having nonzero means and different variances. Projected residuals (Cook and Tsai, 1985) overcome these shortcomings, as they have zero means and are uncorrelated with predicted values.
(TIF)
