# Correction: Continuing a cancer treatment despite tumor growth may be valuable: Sunitinib in renal cell carcinoma as example

**DOI:** 10.1371/journal.pone.0105179

**Published:** 2014-07-31

**Authors:** The PLOS ONE Staff

The legend for Figure 2 is incorrect. Figure 2 and its complete, correct legend can be seen here.

**Figure 2:**
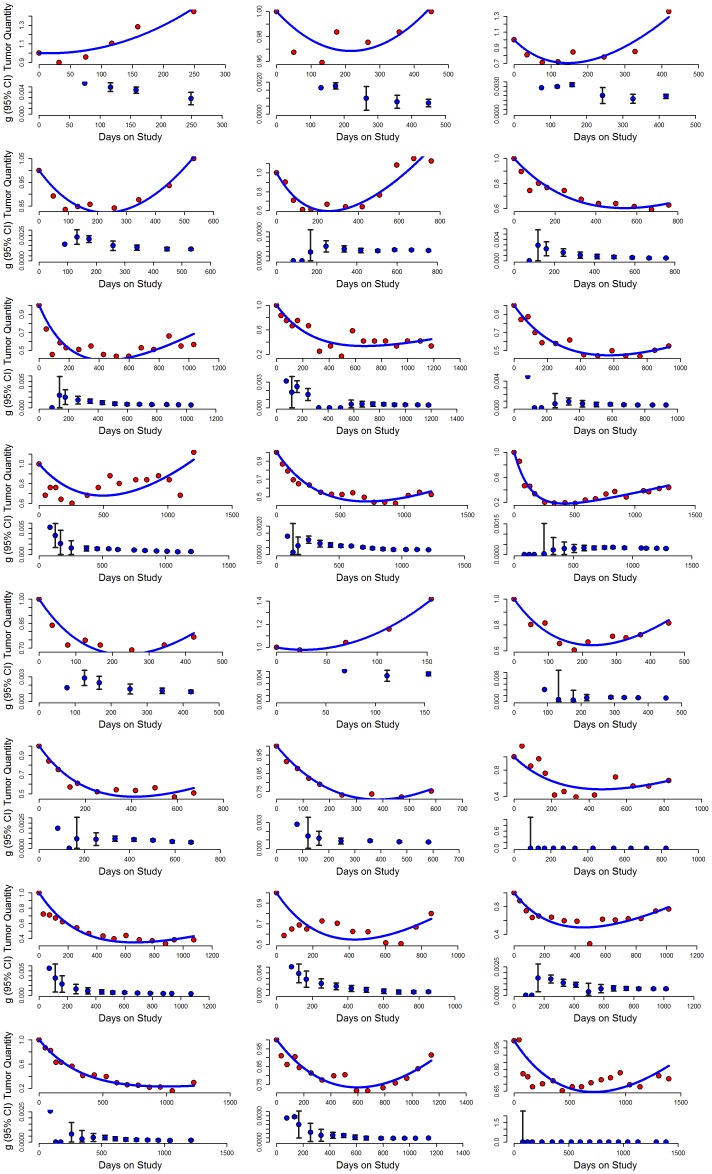
Observed and predicted values of tumor quantity over time in 24 patients randomized to sunitinib in the registration trial. The majority of patients randomized to sunitinib had no evidence of acceleration in the rate of growth for hundreds of days. The majority had stable rates of growth as shown above; some had only evidence of tumor regression, but these are not shown. In each example, the upper graph plots the observed tumor quantity measurements obtained by the clinical investigators during the patient's participation in the clinical trial as well as the predicted values from the best-fit model. The lower panel of each pair depicts the growth rate constant, g, calculated with the data gathered up to that point in time, showing serial calculations of this value. The first calculation is done when three data points had been obtained, and each point thereafter depicts the estimate and 95% CI of the growth rate constant as each new data point is obtained.
